# Analysis of Toll-Like Receptors, iNOS and Cytokine Profiles in Patients with Pulmonary Tuberculosis during Anti-Tuberculosis Treatment

**DOI:** 10.1371/journal.pone.0088572

**Published:** 2014-02-18

**Authors:** Larissa Ragozo Cardoso de Oliveira, Eliana Peresi, Marjorie de Assis Golim, Mariana Gatto, João Pessoa Araújo Junior, Érika Alessandra Pellison Nunes da Costa, Jairo Aparecido Ayres, Maria Rita Parise Fortes, Sueli Aparecida Calvi

**Affiliations:** 1 Tropical Diseases Department, Botucatu School of Medicine – UNESP, Botucatu, São Paulo, Brazil; 2 Flow Cytometry Laboratory, Hemocenter, Botucatu School of Medicine – UNESP, Botucatu, São Paulo, Brazil; 3 Department of Microbiology and Immunology, Biosciences Institute, UNESP, Botucatu São Paulo, Brazil; 4 Nursing Department, Botucatu School of Medicine – UNESP, Botucatu, São Paulo, Brazil; 5 Dermatology and Radiotherapy Department, Botucatu School of Medicine – UNESP, Botucatu, São Paulo, Brazil; The Ohio State University, United States of America

## Abstract

Toll-like receptors (TLRs) play an important role in mycobacterial infection, although little is known about the roles of these receptors, cytokines and nitric oxide during anti-tuberculosis treatment. Our objective was to evaluate the mRNA and cell surface expression of TLR2 and TLR4; inducible nitric oxide synthase (iNOS) expression; and cytokine Th1, Th2 and Th17 profiles in pulmonary tuberculosis patients at different time points of anti-tuberculosis treatment. Peripheral blood mononuclear cells (PBMCs) were obtained from PPD^+^ healthy controls and from patients receiving anti-tuberculosis treatment. Gene expression quantification was performed by qPCR, cell surface expression was assessed using flow cytometry, and cytokine quantification was conducted using the CBA technique. The treated patients presented higher gene expression and higher numbers of receptors on the cell surface of lymphocytes and monocytes than did control individuals. IL-12 and IFN-γ levels increased after the start of treatment, whereas TNF-α levels were reduced. TGF-β presented the highest levels during treatment. IL-10 and IL-17 expression and production tended to increase during treatment. *iNOS* gene expression was reduced throughout treatment in patients. Our results suggest that anti-tuberculosis treatment modulates the immune response, inducing an increase in the expression of TLRs and pro- and anti-inflammatory cytokines to combat bacteria and reduce the inflammatory process.

## Introduction

Tuberculosis (TB) is an infectious disease with chronic evolution, and its etiological agent is the intracellular bacterium *Mycobacterium tuberculosis* (*M. tuberculosis*) [1]. Toll-like receptor (TLR) 2 is the main receptor for mycobacterial constituents, recognizing lipoarabinomannan (LAM); its precursor, phosphatidylinositol mannoside (PIM); and 19-kDa lipoprotein [2]–[6]. TLR4 is a receptor for exogenous ligands, such as LPS from Gram-negative bacteria, and can recognize endogenous ligands, such as heat shock protein (HSP) 60/65, which is released by mycobacteria [7], [8]. Studies have shown that the recognition of mycobacterial products by TLRs leads to NF-κB activation and consequently to gene transcription that produces pro-inflammatory cytokines, such as IL-12, TNF-α, IL-1β and nitric oxide [9].

The recognition of *M. tuberculosis* by TLRs induces phagocytosis by alveolar phagocytes and the production of IL-12 by macrophages and dendritic cells. IL-12 stimulates natural killer (NK) cells and Th1 responses that produce IFN-γ [10], [11]. IFN-γ is responsible for activating macrophages to produce TNF-α, which, in synergy with IFN-γ, acts to increase phagocytosis and microbicidal activity through the production of reactive nitrogen and oxygen intermediates involved in the growth inhibition and death of mycobacteria [12]–[15]. TNF-α is also essential for forming and maintaining granulomas [16], [17], [12]. Studies have suggested that protective immunity against *M. tuberculosis* and Th1 responses require Th17, mainly at the start of infection [18]. IL-17 has pro-inflammatory properties that induce the expression of cytokines, chemokines and metalloproteinases, which are important in neutrophil recruitment, activation and migration [19]. Despite the protective effect of Th1 and Th17 responses against tuberculosis, the elevated expression of pro-inflammatory cytokines is related to disease immunopathogenesis [20], [21]. To limit this deleterious action, anti-inflammatory mechanisms arise, represented by soluble TNF-α receptors that impede this cytokine's binding to its receptor through signal blockade by regulatory T cells and the anti-inflammatory cytokines IL-4, IL-10 and TGF-β [21]–[23].

Studies have shown that TLRs regulate the intracellular destination of bacteria through a complicated cascade of regulators and deregulators. However, the roles of TLRs, cytokines and nitric oxide during anti-tuberculosis treatment are unknown. In light of these observations, studies evaluating TLRs; inducible nitric oxide synthase (iNOS); and Th1, Th2 and Th17 cytokines in patients during anti-tuberculosis treatment may contribute to a better understanding of the host/pathogen relationship in this disease. Our study evaluated the mRNA and cell surface expression of TLR2 and TLR4; iNOS expression; and the production and expression of IL-12, IFN-γ, TNF-α, IL-17, IL-10 and TGF-β in pulmonary tuberculosis patients during anti-tuberculosis treatment.

## Materials and Methods

### Patients

The study recruited 20 healthcare workers (C) as controls (13 males and six females; mean age of 49 and 37 years, respectively) with a positive tuberculin skin test (TST) (induration ≥10 mm) from Botucatu Medical School University Hospital. These individuals had no clinical complaints or with no history of TB disease, autoimmune disease or other infectious disease. Samples from C were collected at one time point. The study also included 19 pulmonary tuberculosis patients (TB) (13 males and six females; mean age of 49 and 37 years, respectively) treated at the Infectious and Parasitic Diseases Services at Botucatu Medical School University Hospital – UNESP, Botucatu Teaching Health Centre, and at Primary Healthcare units in Botucatu and the surrounding region. Tuberculosis patients were diagnosed based on a sputum smear or culture positive for *M. tuberculosis* or clinical-epidemiologic data and laboratory and imaging exams compatible with active tuberculosis. Patients with pulmonary tuberculosis concurrent with other active granulomatous disease, autoimmune disease, cancer, HIV or other immunodeficiency or multidrug resistance and pregnant women were excluded. All pulmonary tuberculosis patients were treated for six months using the standard scheme (rifampicin, isoniazid, pyrazinamide and ethambutol). Samples from patients were collected based on the anti-tuberculosis treatment timeline: **M1:** patients with no more than one month of anti-tuberculosis treatment; **M2:** in the third month of anti-tuberculosis treatment; and **M3:** at the end of six months of anti-tuberculosis treatment.

All of the patients and controls agreed to participate in the study after due clarification and signing of a written informed consent form. This study was approved by Botucatu Medical School – UNESP Research Ethics Committee.

### Blood sample collection

Blood samples (20 ml) were collected from the forearm vein at one time point from controls and at three different time points of anti-tuberculosis treatment from pulmonary TB patients. Samples were collected in heparinized tubes and initially centrifuged at 1,500 g for 10 minutes to obtain plasma for measuring cytokines by CBA. The remaining blood samples were used to obtain peripheral blood mononuclear cells (PBMCs) for later evaluation of the gene expression of *TLR2*, *TLR4*, *iNOS* and cytokines and the expression of TLR2 and TLR4 on the cell surface using flow cytometry.

### Harvesting mononuclear cells from peripheral blood

PBMCs were obtained by the Histopaque® gradient separation method [24]. The layer rich in lymphocytes and monocytes was aseptically removed and washed with PBS for 15 minutes at 450 g. The cells were then resuspended in PBS. Cell identification and viability analysis were performed by Turk count. A 1×10^6^/ml or 2×10^6^/ml cell concentration was then prepared for the described protocols.

### *TLR2*, *TLR4*, *IL-12*, *IFN-γ*, *TNF-α*, *IL-17*, *IL-10*, *TGF-β* and *iNOS* mRNA expression

Total RNA was extracted from PBMCs at 2×10^6^ cells/ml that were obtained once from controls or at M1, M2 and M3 of anti-tuberculosis treatment from pulmonary TB patients by the TRIzol method (Gibco-BRL, São Paulo, Brazil). The RNA concentration was determined by absorbance at 260 nm; all samples showed an absorbance value of approximately 2.0. One microgram of RNA was used for the synthesis of 20 µL of complementary DNA (cDNA) by SuperScript™ III Reverse Transcriptase (Invitrogen, São Paulo, Brazil). *TLR2*, *TLR4*, *IL-12*, *IFN-γ*, *TNF-α*, *IL-10*, *TGF-β*, *IL-17* and *iNOS* mRNA levels were determined by real-time PCR. Primer sequences are shown in Table 1. The *β-actin* gene was used as an internal control. Relative quantification of each target mRNA was performed using a standard curve-based method for relative real-time PCR data processing with a 7300 Real-Time PCR System (Applied Biosystems, USA) and Power SYBR Green PCR Master Mix [25]. The real-time PCR cycler conditions were 95°C for 10 minutes, followed by 40 annealing cycles at 95°C for 15 seconds and extension at 60°C for 1 minute. Fluorescence signals were collected during the annealing and extension cycle of amplification (60°C for 1 minute). Amplification of specific transcripts was confirmed based on the melting curve profiles generated at the end of each run. The control samples' mean expression was assigned a relative value of 1.0, and concentrations in all other samples were normalized proportionately.

**Table 1:** Primers for cytokines, iNOS, β-actin and TLRs.

Gene	Reverse Sequence	Forward Sequence	GenBank	Product Length
**IL-12p40**	5′-CTCCTGCCTCATCCTCCTGAA-3′	5′-CAGCCTGGGAAACATAACAAGAC-3′	NM_002187.2	109
**IFN-γ**	5′-GTTCCATTATCCGCTACATCTGAA-3′	5′-AGCTCTGCATCGTTTTGGGTT-3′	NM_000619.2	118
**TNF-α**	5′-GATGATCTGACTGCCTGGGC-3′	5′-CACGCTCTTCTGCCTGCTG-3′	NM_000594.2	105
**IL-17**	5′-GGATTTCGTGGGATTGTGAT-3′	5′-TGGGAAGACCTCATTGGTGT-3′	NM_002190.2	84
**IL-10**	5′-TCATCTCAGAACAAGGCTTGGC-3′	5′-CGAGATGCCTTCAGCAGAGTG-3′	NM_000572.2	128
**TGF-β**	5′-TCCAGGCTCCAAATGTAGG-3′	5′-GGACACCAACTATTGCTTCAG-3′	NM_000660.4	150
**TLR2**	5′-GGTCTTGGTGTTCATTATCTTC-3′	5′-TCTCCCATTTCCGTCTTTTT-3′	NM_003264.3	125
**TLR4**	5′-TCTGCTGCAACTCATTTCAT-3′	5′-CCGCTTCCTGGTCTTATCAT-3′	NM_138554.3	141
**iNOS**	5′-GCGTTACTCCACCAACAATGGCAA-3′	5′-ATAGAGGATGAGCTGAGCATTCCA-3′	NM_000625.4	109
**β-actin**	5′-AAGGGACTTCCTGTAACAATGCA-3′	5′-CTGGAACGGTGAAGGTGACA-3′	NM_001101.3	140

### TLR2 and TLR4 cell surface expression

PBMCs obtained and adjusted to a concentration of 1×10^6^ cells/ml were centrifuged at 650 g for 10 minutes at 4°C. The supernatant was then discarded, and the cells were incubated with a monoclonal anti-TLR4 antibody conjugated to PE, an anti-TLR2 antibody conjugated to FITC, an anti-CD3 antibody conjugated to PE-DY647 and an anti-CD14 antibody conjugated to PE-DY647 (BioLegend, Brazil) for 20 minutes in a dark environment. After incubation, the cells were resuspended in electrolyte solution (ISOTON II) and fixed in a fixer solution containing 5% formaldehyde (Becton, Dickinson and Company). Analyses were performed using flow cytometry (FACSCalibur™, Becton Dickinson) using CellQuest software (Becton Dickinson) for cell acquisition and analysis. Acquisition was standardized for 10,000 events per sample. Each test contained a control tube in which cells were incubated with isotopic control antibodies labeled with respective fluorochromes.

### Cytokine production

The levels of the cytokines IL-12, IFN-γ, TNF-α, IL-17, TGF-β and IL-10 were measured in the plasma by the CBA technique and analyzed using flow cytometry (FACSCalibur™, Becton Dickinson) using CellQuest software (Becton Dickinson) according to the manufacturer's instructions.

### Statistical analysis

The gene expression of *TLR2*, *TLR4*, *IL-12*, *IFN-γ*, *TNF-α*, *IL-10* and *TGF-β* was compared using ANOVA in repeated-measurement design on time, followed by an adjusted Tukey's test for multiple comparisons when the data presented a normal distribution. In the case of a non-normal distribution of data on TLR2, TLR4, IL-12, IFN-γ, TNF-α, IL-10, TGF-β and IL-17 levels in patients and controls, the same design was fitted using a generalized linear model with a gamma distribution. Differences in values for sputum smears between groups of patients were assessed using a Kruskal-Wallis test with Dunn's post-test All of the analyses were performed using SAS for Windows software, V.9.2. The results were considered significant when p<0.05.

## Results

### Sputum smears

**Figure 1:**
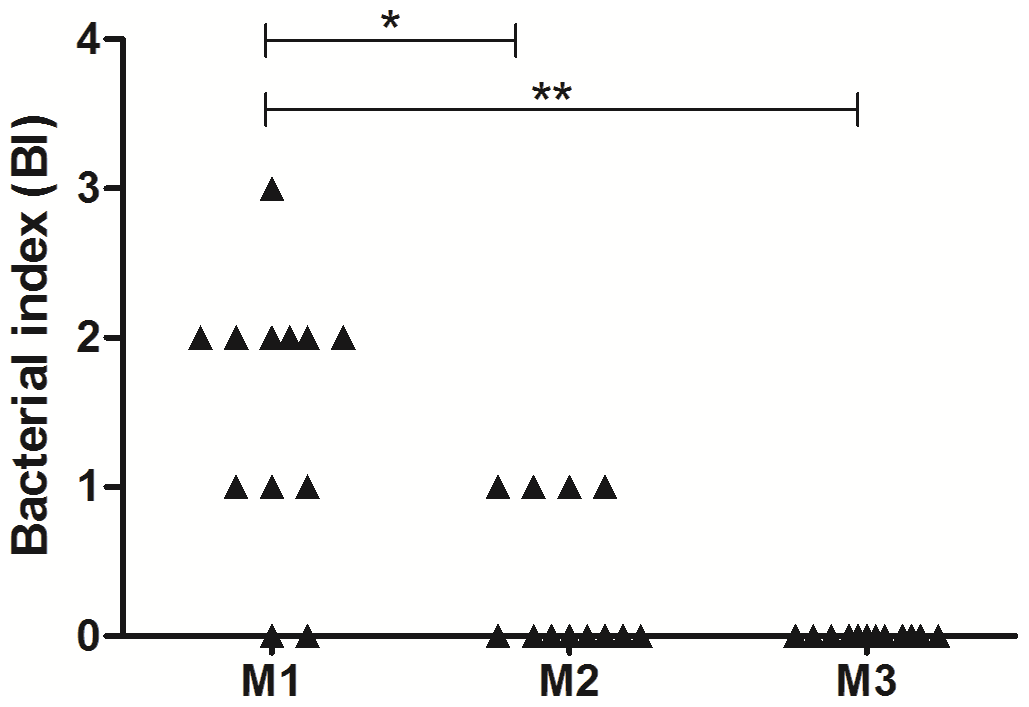
Bacterial index. Bacterial index of pulmonary tuberculosis patients (G2) during anti-tuberculosis treatment and at M1 (up to one month of treatment), M2 (three months of treatment) and M3 (end of treatment). M1: 0, N = 2; +1, N = 3; +2, N = 6; +3, N = 1. M2: 0, N = 8; +1, N = 4. M3: 0, N = 12. *p = 0.05 M1 vs. M2, **p = 0.05 M1 vs. M3.

Sputum smears were evaluated using the bacteriological index (BI) on Ridley's logarithmic scale, ranging from zero to four. The results showed a significant decrease in bacterial load during treatment (M1–M2, p = 0.05; M1–M3, p = 0.01) (Figure 1).

### *TLR2* and *TLR4* gene expression

**Figure 2:**
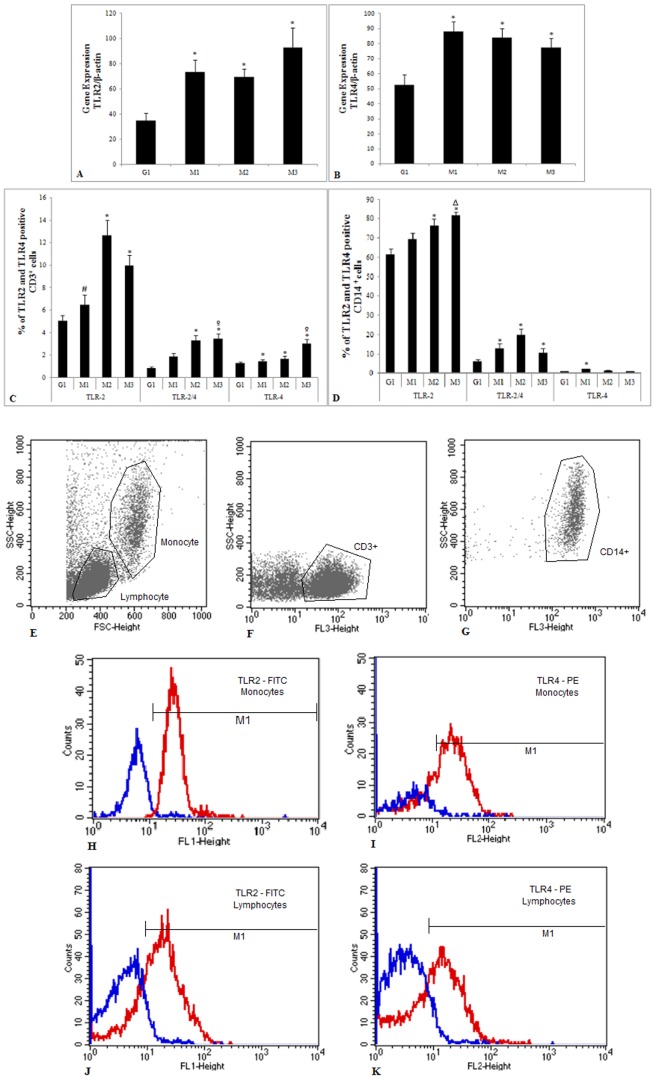
Gene expression and cell surface of TLR2 and TLR4. mRNA expression of TLR2 (A) and TLR4 (B) and mean percentages of CD3^+^ (C) and CD14^+^ (D) cells expressing TLR2, co-expressing TLR2 and TLR4 and expressing TLR4 among PBMCs from control individuals (G1) and pulmonary tuberculosis patients (G2) during anti-tuberculosis treatment at M1 (up to one month of treatment), M2 (three months of treatment) and M3 (end of treatment). Gating strategies to distinguish between PBMC populations analyzed by flow cytometry. FSC-SSC profile was used to distinguish total lymphocytes and monocytes (E). T lymphocytes and monocytes were gated according to light scatter profile and the expression of CD3 (F) and CD14 (G), respectively. Representative histograms plots of TLR2 and TLR4 expression are shown in figures H–K, in which blue histograms represent isotype controls and red histograms indicate TLR-specific antibodies with the respective expression of TLR2^+^ and TLR4^+^ in monocytes (H, I) and T lymphocytes (J, K). The results are expressed as mean and standard deviation and are representative of three independent replicates. *p<0.05 compared with G1; #p<0.05 compared with M2 and M3; ^○^p<0.05 compared with M1 and M2; Δp<0.05 compared with M1.

No significant differences were detected in *TLR2* gene expression between patients at different treatment time points (M1, M2 and M3). *TLR2* gene expression in patients during treatment (M1, p = 0.003; M2, p = 0.003; and M3, p = 0.0002) was significantly higher than in control individuals. The gene expression of this receptor tended to increase during treatment (Figure 2A). *TLR4* gene expression was significantly higher in pulmonary tuberculosis patients (TB) at the different time points of treatment (M1, p = 0.01; M2, p = 0.01; M3, p = 0.04) than in controls (C) (Figure 2B). No significant differences were detected in *TLR4* gene expression between patients at different treatment time points (M1, M2 and M3). The gene expression of this receptor tended to diminish during treatment (Figure 2B).

### TLR2 and TLR4 surface expression

The expression and co-expression of TLR2 and TLR4 on monocyte and lymphocyte cell surfaces was evaluated by measuring the percentage of CD3^+^ and CD14^+^ cells that were positive for TLR2 and TLR4 during treatment. The surface expression of TLR2 on lymphocytes (Figure 2C) in patients was significantly lower at M1 than at M2 (p = 0.006) and M3 (p = 0.04). TB patients presented a significant increase in the expression of this receptor at M2 (p<0.0001) and M3 (p<0.0001) compared with controls. At all three treatment time points in TB patients, the expression of TLR4 (M1, p = 0.0019; M2, p<0.0001; M3, p<0.0001) and the co-expression of TLR2/4 (M1, p = 0.0006; M2, p<0.0001; M3, p<0.0001) on lymphocyte cell surfaces were significantly higher than in the controls and tended to increase during treatment. TLR4 expression was also significantly higher at M3 than at M1 (p = 0.001) and M2 (p<0.0001). The co-expression of TLR2/4 at M3 was significantly higher than at M1 (p = 0.02) (Figure 2C).

Monocyte analysis (Figure 2D) showed increased TLR2 expression in TB patients at M1 (not significant), M2 (p = 0.004) and M3 (p<0.0001) compared with controls. Expression was also significantly higher at M3 than at M1 (p = 0.004). The co-expression results for these receptors on monocyte cell surfaces also showed a significant increase in TB patients at all time points of treatment (M1, p = 0.005; M2, p<0.0001; and M3, p = 0.01) compared with controls. The expression of TLR4 in TB patients at M1 (p = 0.0019) was significantly higher than in controls. Interestingly, cells labeled with anti-CD3 and anti-CD14 showed much higher TLR2 than TLR4 expression, including both monocytes and lymphocytes, and the majority of cells expressing TLR4 also expressed TLR2.

### Cytokine mRNA expression and production

**Figure 3:**
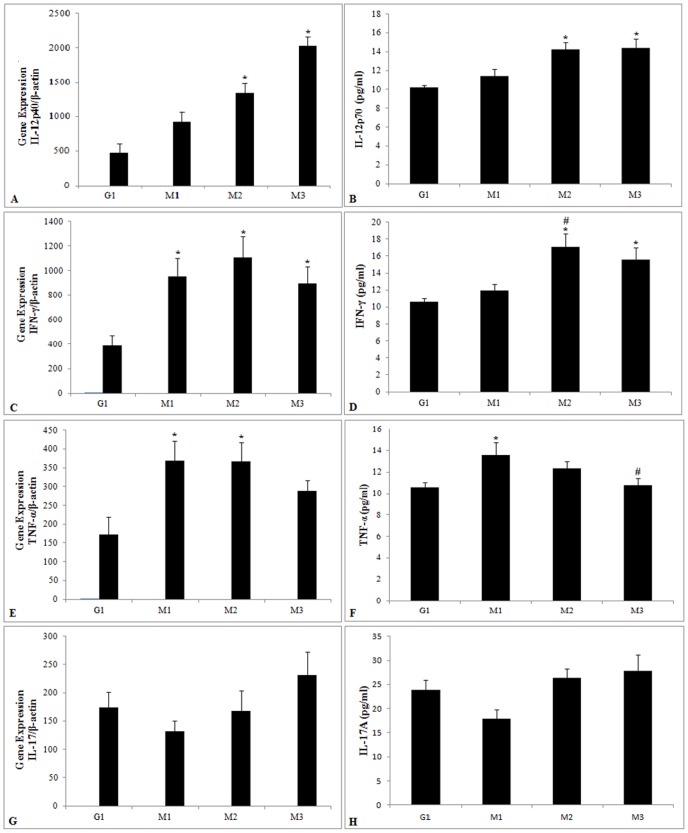
Expression and production cytokines inflammatory. mRNA expression and production of IL-12 (A, B), IFN-γ (C, D), TNF-α (E, F) and IL-17 (G, H) in the control group (G1) and in the patient group during anti-tuberculosis treatment (G2) and at M1 (up to one month of treatment), M2 (three months of treatment) and M3 (end of treatment). The results are expressed as the mean and standard deviation and are representative of three independent replicates. *p<0.05 compared with G1; #p<0.05 compared with M1.

In TB patients, IL-12 mRNA expression and production tended to increase during treatment. Significantly higher levels of mRNA expression were detected at M2 and M3 (p = 0.005 and p = 0.001, respectively) in relation to expression in controls. Increased IL-12 production levels were observed in TB patients at M2 and M3 (p<0.0001 and p = 0.0009, respectively) in relation to controls (Figures 3A and B).

Regarding *IFN-γ* gene expression and production, treatment induced significantly higher levels of this cytokine at M2 (p = 0.02) and M3 (p = 0.001), and gene expression presented higher levels at M1 (p = 0.0003), M2 (p<0.0001) and M3 (p = 0.001) compared with expression in control individuals. No significant differences were detected in mRNA expression for *IFN-γ* during treatment, and production was significantly higher at M2 in relation to M1 (p = 0.02). Both the gene expression and production of *IFN-γ* tended to decrease at M3 (Figures 3C and D).

An analysis of *TNF-α* gene expression and production (Figures 3E and F) showed significantly increased expression at M1 (p = 0.03) and M2 (p = 0.009) and increased production at M1 (p = 0.005) in TB patients compared with controls. No significant differences were detected in *TNF-α* expression between different treatment time points. *TNF-α* production was lower at M3 (p = 0.01) than at M1.

*IL-17* gene expression and production in TB patients did not present any significant differences in relation to the control group. There were also no differences between the different treatment time points (M1, M2 and M3) (Figures 3G and H).

**Figure 4:**
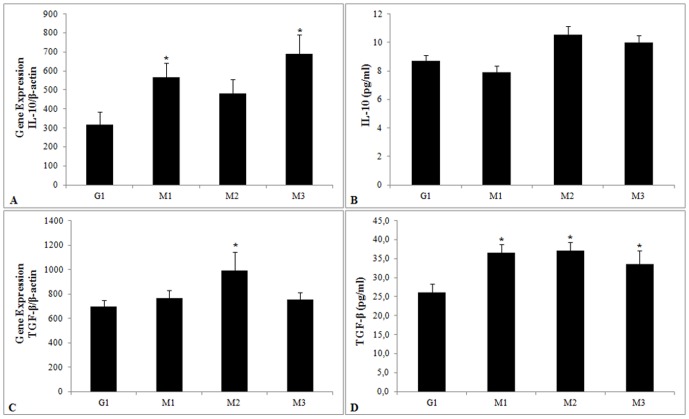
Expression and production cytokines IL-10 and TGF-β. mRNA expression and production of IL-10 (A, B) and TGF-β (C, D) in the control group (G1) and in the patient group during anti-tuberculosis treatment (G2) and at M1 (up to one month of treatment), M2 (three months of treatment) and M3 (end of treatment). The results are expressed as the mean and standard deviation and are representative of three independent replicates. *p<0.05 compared with G1.

*IL-10* gene expression in TB patients was significantly higher at M1 (p = 0.01) and M3 (p = 0.01) than in controls; *IL-10* production in TB patients did not present any significant differences in relation to the control group. There were also no differences between the different treatment time points (M1, M2 and M3) (Figures 4A and B).

*TGF-β* gene expression in TB patients was significantly higher at M2 (p = 0.006) in relation to controls. TGF-β production showed a significant increase in expression at M1 (p<0.0001), M2 (p = 0.001) and M3 (p = 0.01) in relation to controls (Figures 4C and D).

### *iNOS* expression

**Figure 5:**
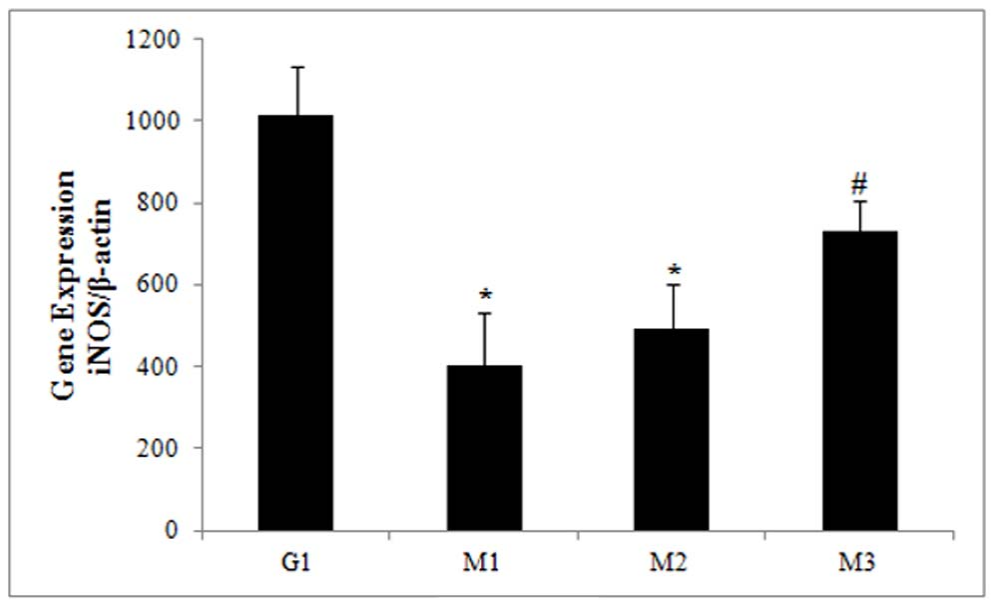
*iNOS* expression. mRNA expression of *iNOS* in the control group (G1) and the patient group during anti-tuberculosis treatment (G2) and at M1 (up to one month of treatment), M2 (three months of treatment) and M3 (end of treatment). The results are expressed as the mean and standard deviation and are representative of three independent replicates. *p<0.05 compared with G1; #p<0.05 compared with M1.

Induction of *iNOS* gene expression in TB patients was significantly lower at M1 (p = 0.01) and M2 (p = 0.03) than in controls (G1) (Figure 5). Additionally, the expression of this enzyme was higher at M3 (p = 0.001) than at M1.

## Discussion

In this study, we evaluated the gene and cell surface expression of TLR2 and TLR4 in pulmonary tuberculosis patients during anti-tuberculosis treatment. We showed that these patients exhibit both receptors and expression increases during anti-tuberculosis treatment. We also found that when cells were labeled with anti-CD3 and anti-CD14, both lymphocyte and monocyte populations presented higher TLR2 expression than TLR4 expression, and most cells that expressed TLR4 also expressed TLR2. These results are in agreement with the findings of other studies demonstrating that TLR2 and TLR4 are involved in *M. tuberculosis* recognition [26]–[28]. Our results showed that although receptor expression is higher in monocytes, this expression is also observed in lymphocytes. These results are in agreement with the findings of other studies that have shown increases in TLR mRNA expression in CD4^+^ and CD8^+^ T lymphocytes in acute tonsillitis [29] and in various lymphocyte subtypes TB patients' pleural fluid [30].

TLR ligands have different effects on innate immune cells, such as monocytes, including the induction and production of cytokines, the expression of costimulatory molecules and the expression of MHC II molecules. Studies in mice with genes from inactivated TLRs have shown that TLR2 expression in monocytes is important in infection control and survival in these animals. Other studies have suggested a protective role for TLR4 expression in monocytes in mouse survival [31]–[34], depending on the *Mycobacterium* dose [34]. In T lymphocytes, these receptors can act as costimulatory receptors for the TCR, increasing the proliferation of stimulated T cells and/or the production of cytokines. Different antigens from mycobacteria can indirectly modulate T cell function through functional changes in antigen-presenting cells, although direct interactions between *M. tuberculosis* molecules and T cells can occur when mycobacterial components contained in vesicles are liberated by infected macrophages [35]–[37]. Differences between expression and production can be explained by mRNA stability, the transcription rate and factors that regulate translation that can directly affect the expression and production of mediators involved in immune responses [38].

High TLR2 and TLR4 expression during anti-tuberculosis treatment associated with a moderate form of disease suggests that these receptors were seems likely beneficial to the patients because such TLRs can induce the production of pro-inflammatory cytokines. In this sense, we showed that pulmonary tuberculosis patients at the start of the treatment presented similar IL-12 gene expression levels and production as did controls, and these parameters increased during anti-tuberculosis treatment. Sahiratmadja et al (2007) [39] showed that after two months of similar treatment, IL-12 levels considerably increased, becoming higher than levels in controls [39]. Contrary to what we observed, others have shown that serum levels of IL-12p40 were not higher in patients with active tuberculosis during anti-tuberculosis treatment than in healthy controls or contactants [40]. A possible explanation for these results could be differences in experimental protocols, such as the treatment periods evaluated and the cytokine detection techniques. IL-12 is important in mediating protective immunity against TB and is induced following phagocytosis of *M. tuberculosis* by macrophages and dendritic cells [41], which leads to the development of a Th1 response, with production of IFN-γ [41].

Our study showed significantly increased mRNA expression for IFN- γ in TB patients at the beginning of treatment, and plasma levels tended to increase during treatment in relation to control individuals. Additionally, protein expression and production, but mainly production, increased at the three months of treatment and tended to decrease at the end of treatment. A study showed that recently diagnosed patients presented higher serum IFN-γ levels than did individuals with a previous tuberculosis history, although the levels were similar to those of healthy controls. In the same study, untreated individuals presented much higher levels of this cytokine, which gradually decreased during treatment [42]. These results differ from ours and those of another study in which IFN- γ was shown to increase during the course of treatment [39]. A possible explanation for this cytokine's increased levels after treatment is based on a study performed by Jo et al. (2003) [43], which showed that at the moment of tuberculosis diagnosis, the IFN-γ concentration is much higher at the infection site than in the peripheral blood, suggesting that during tuberculosis activity, there is a localization of specific lymphocytes against the mycobacterial antigen at the infection site and that the increase in the IFN-γ serum concentration at the chemotherapy stage coincides with the arrival of lymphocytes in the peripheral blood. In contrast to our findings, other studies have observed that IFN- γ levels are depressed in patients with active tuberculosis [44], [45]. Patients with the moderate form of TB present higher IFN-γ levels than patients with the more severe form [39]. Although our results showed that the expression of this cytokine was higher at the start of treatment, this elevated expression did not translate into production, which only showed a trend toward being higher than in control individuals. Levels in this phase of treatment were not related to protection, seems likely because quantities were still not adequate to activate the mechanism responsible for mycobacteria destruction because the bacterial load was high in most patients at this stage. IFN- γ levels increased during treatment and were seems likely sufficient to activate a protective response.

TNF-α, an important cytokine for infection control, is involved in the macrophage activation process and is also an important factor related to disease immunopathology [46]. In our study, tuberculosis patients presented significantly higher TNF-α levels than did controls. During treatment, patients presented gradually decreasing levels of this cytokine. Other studies have also shown that individuals with tuberculosis presented elevated TNF-α levels in PBMC culture supernatant compared with controls, and these levels decreased during treatment [46], [42]. In our study, despite the high TNF-α levels in patients at the start of treatment, we observed that this cytokine was not protective at this phase and could have been involved in disease pathogenesis. Because treatment decreased TNF-α levels, we suggest that at lower levels, this cytokine could be involved in protection through the stimulation of pathogenic mechanisms. TNF-α, depending on the concentration produced, could be involved in immunopathological effects such as fever, body weight reduction, tissue necrosis and shock [47]–[55].

In our study, pulmonary tuberculosis patients tended to present much lower IL-17 levels at the start of treatment compared with controls. During treatment, production and expression tended to increase. Th17 cells, which are involved in the development of inflammatory and autoimmune diseases, are also involved in protection against certain intracellular pathogens [56]–[58], including *M. tuberculosis* [59], [60]. However, the exact role of Th17 cells in individuals with pulmonary tuberculosis, mainly during anti-tuberculosis treatment, is not very clear [61]. IL-17 can be induced immediately after pulmonary infection with BCG [57] and can also be detected in the later stages (4–52 weeks) of *M. tuberculosis* infection [60]. The frequency of Th17 cells in pulmonary TB patients has been reported as significantly lower than in healthy controls and individuals with latent TB [56]. These results suggest that a reduced Th17 response could be associated with the clinical manifestation of pulmonary TB and that this cell subtype might be involved in protection, rather than disease immunopathogenesis. These ideas agree with our findings, as patients at the start of treatment had low IL-17 levels that tended to increase with treatment and pathogen killing.

Our results showed that the production of anti-inflammatory cytokines, such as IL-10 and TGF-β, tended to rise during anti-tuberculosis treatment and to diminish at the end of treatment. This phenomenon suggested that these cytokines' main actuation was at the end of treatment, exerting a regulatory role to control the inflammatory process. Other human studies on tuberculosis have suggested that IL-10 also has a critical role in protecting the host against inflammatory immunopathology [62]. In contrast to our results, studies have shown that patients with a recent diagnosis of pulmonary tuberculosis present higher serum levels of IL-10 than do previously treated or healthy individuals, although treatment reduces the serum concentration of this cytokine [40], [42]. Moreover, another study observed that before treatment, tuberculosis patients presented similar levels of this cytokine as controls [63]. We observed differences related to production and expression during treatment. Differences between expression and production can be explained by mRNA stability, the transcription rate and factors that regulate translation that can directly affect the expression and production of mediators involved in immune responses [38].

In tuberculosis, TGF-β can primarily exert a suppressive role as part of a Th1 profile and participate in fibrosis induction [46]. At low concentrations, this cytokine still acts as a chemotactic factor for monocytes, inducing IL-1α and TNF-α secretion and participating in Th17 cell differentiation, together with IL-6 and IL-21 [64], and Treg cell differentiation [65]. Our results agree with the literature, which reports that patients with pulmonary TB do not present a deficiency in TGF-β production in active disease or during anti-tuberculosis treatment [46], [63]. During treatment, we suggest that the high levels of this cytokine are regulating inflammatory activity, contributing to protecting against the damage caused by the exacerbated inflammatory response and participating in extracellular matrix deposition and fibrotic processes.

NO is considered to be one of the main mediators involved in *Mycobacterium* killing [66], and NO generation is dependent on the iNOS enzyme [67]. To our knowledge, this is the first study to evaluate iNOS in pulmonary tuberculosis patients during anti-tuberculosis treatment. We observed an decrease of the gene expression of this enzyme during treatment compared with expression in control individuals. Certain studies have suggested that the inhibition of *iNOS* expression and NO production can be considered as an escape mechanism for various infectious agents, such as *Mycobacterium leprae* and *M. tuberculosis* [68]. Certain *M. tuberculosis* antigens, such as CFP-10 and 19-kDa protein, can affect macrophage function, inhibiting macrophages' microbicidal capacity and creating a favorable environment for *M. tuberculosis* survival [69]. The mycobacterial cell wall component LAM can directly inhibit phagolysosome fusion [70], [71], and studies have suggested that *Mycobacterium* can impede its recruitment to the phagolysosome, also characterizing an escape mechanism [72]. Another fact that must be taken into account is that other microbicidal mechanisms, such as oxygen metabolites, can be important in bacteria killing, including the superoxide anion and hydrogen peroxide [73]. Because our results did not show an association between TLRs and cytokines, we were not able to confirm that the levels of cytokines and iNOS measured in the study subjects were dependent on TLR2 and TLR4. Our results also lack an association between demographic characteristics and expression and production of the variables evaluated. These results may be due to our small sample size, high standard variation and the fact that all patients had a moderate presentation of PTB.

Our study showed that during anti-tuberculosis treatment, pulmonary tuberculosis patients presented increased TLR expression and pro- and anti-inflammatory cytokine levels, which were seems likely responsible for controlling infection and excess inflammation. Therefore, we suggest that during anti-tuberculosis treatment, mycobacteria killing could occur due to a direct effect of the treatment, as well as by the activation of several mediators of the immune response.
